# Soluble and Membrane-Bound TGF-β-Mediated Regulation of Intratumoral T Cell Differentiation and Function in B-Cell Non-Hodgkin Lymphoma

**DOI:** 10.1371/journal.pone.0059456

**Published:** 2013-03-15

**Authors:** Zhi-Zhang Yang, Deanna M. Grote, Steven C. Ziesmer, Bing Xiu, Nicole R. Yates, Frank J. Secreto, Lucy S. Hodge, Thomas E. Witzig, Anne J. Novak, Stephen M. Ansell

**Affiliations:** 1 Division of Hematology, Mayo Clinic College of Medicine, Mayo Clinic, Rochester, Minnesota, United States of America; 2 Department of Hematology, Tongji Hospital, Tongji University, Shanghai, China; New York University, United States of America

## Abstract

While the effect of TGF-β on malignant B cells in non-Hodgkin lymphoma (NHL) has been previously evaluated, studies to specifically define the role of TGF-β in tumor immunity in B-cell NHL are limited. We found that soluble TGF-β, secreted by both lymphoma cells and intratumoral T cells, is present in the serum of patients with B-cell NHL. Soluble TGF-β promoted regulatory T (T_reg_) cells by enhancing expression of Foxp3 in CD4^+^ T cells and suppressed effector helper T (T_H_) cells by inhibiting expression of IFN-γ and IL-17. Blockade of the IL-2 signaling pathway diminished the effect of soluble TGF-β on T cell differentiation. Furthermore, we found that membrane-bound TGF-β is expressed specifically on the surface of malignant B cells in B-cell NHL. TGF-β was able to bind to the surface of lymphoma B cells through an interaction with heparan sulfate (HS) but not through the TGF-β receptor. We showed that pretreatment of lymphoma B cells with TGF-β significantly inhibits the proliferation and cytokine production of intratumoral T cells. Taken together, these results suggest that tumor-associated soluble and membrane-bound TGF-β are involved in the regulation of intratumoral T cell differentiation and function in B-cell NHL.

## Introduction

Transforming growth factor-beta (TGF-β) is a pleiotropic cytokine that plays a pivotal role in regulating cell growth and differentiation in a variety of cell types [1]. TGF-β can be expressed in a secreted form or be present on the cell surface in a membrane-bound form. Three homologous TGF-β isoforms with additional members form the TGF-β superfamily [1]. TGF-β1 is the predominant isoform expressed in the immune system, but all three isoforms have similar properties in vitro (and will hereafter be referred to collectively as TGF-β). The role of TGF-β in immune response has recently attracted much attention due to the finding that TGF-β is important in the development of T_reg_ and T_H_17 cells [2], [3]. In the malignant scenario, tumor-derived TGF-β suppresses the functions of infiltrating innate and adaptive immune cells, thereby contributing to tumor escape from host immunosurveillance [4].

While soluble TGF-β has been the major focus of previous investigations, recent studies have identified the existence of functional membrane-bound TGF-β, the expression of which is limited to certain subsets of cells including CD4^+^CD25^+^ T_reg_ cells [5], [6]. Membrane-bound TGF-β was found to play a critical role in the CD4^+^CD25^+^ T_reg_ cell-mediated inhibition of CD4^+^CD25^-^ T cells [5] or NK cells [7] through a cell-contact mechanism as well as in the induction of T-cell-mediated tolerance [8]. CD4^+^CD25^-^ T cells expressing membrane-bound TGF-β have been found to significantly suppress the function of other T cells [6], [9]. In addition to CD4^+^ T cells, other types of cells, such as retinal pigment epithelial cells [10], corneal endothelial cells [11], tumor apoptotic bodies [12], head and neck squamous cell carcinoma cells [13] and colorectal cancer cells [14], are able to express membrane-bound TGF-β and inhibit T cell function or induce T_reg_ cell development in a TGF-β-dependent manner.

In B-cell malignancies, both malignant B cells and intratumoral T cells can synthesize and secrete TGF-β. While there is a large body of literature regarding the effects of TGF-β on lymphoma B cells [15], studies regarding the role of TGF-β in tumor immunity in B-cell non-Hodgkin lymphoma (NHL) are very limited. A previous study showed that termination of TGF-β signaling following the transduction of the dominant-negative form of TGF-β receptor II diminished TGF-β-mediated inhibition of EBV-specific cytotoxic cells (CTLs) and enhanced CTL lysis of tumor cells in lymphoma patients [16], [17]. A recent study found that lymphoma T cells trap TGF-β on their cell surface and suppress allogenic T cell function through TGF-β-mediated mechanisms in Sézary patients [18]. These data suggest a potentially significant role for TGF-β in suppressing tumor immunity in B-cell malignancies.

In previous work we have found that an imbalance, favoring an increase in the number of inhibitory T_reg_ cells and a decrease in the number of effector T_H_ cells, exists in the tumor microenvironment of B-cell NHL, which dampens the antitumor immune response [19]–[21]. We have established that malignant lymphoma B cells play a pivotal role in promoting this imbalance [21], [22]. However, the underlying mechanisms by which lymphoma B cells skew the balance between T_reg_ and T_H_ cells are not clear. In the present study, we explored the potential role of TGF-β in mediating a suppressive microenvironment of B-cell NHL. Data generated from this study strongly suggest that TGF-β, in both soluble and membrane-bound form, plays an important role in regulating intratumoral T cell differentiation and function.

## Patients, Materials and Methods

### Patient samples and cell lines

Patients providing written informed consent were eligible for this study if they had a tissue biopsy that upon pathologic review showed B-cell NHL and adequate tissue to perform the experiments. The use of human tissue samples for this study was approved by the Institutional Review Board of the Mayo Clinic/Mayo Foundation (IRB#: 08-004097 Serum cytokines, chemokines, and soluble ligands in non-Hodgkin lymphoma). The biopsy specimens were reviewed and classified using the World Health Organization Lymphoma classification. Forty-four patient samples of different histologies including diffuse large B-cell lymphoma, follicular lymphoma, marginal zone lymphoma, mantle cell lymphoma and small lymphocytic lymphoma were used in this study (Table S1). B cell lines used: DoHH2 (German Resource Centre for Biological Material (DSMZ), Braunschweig, Germany); Karpas 422 (DSMZ); OCI-Ly3 [23], [24]; OCI-Ly10 [23], [24]; OCI-Ly19 [23], [24]; Raji (ATCC); SuDHL1 (DSMZ); SuDHL4 (DSMZ); SuDHL6 (DSMZ); RL (DSMZ); BCWM.1 [25]; MWCL-1 [26]; Mino (DSMZ); Jeko (DSMZ). T cell lines used: Jurkat (DSMZ); HH (ATCC); Karpas 299 [27]; FE-PD [28].

### Cell isolation and purification

CD3^+^, CD4^+^, CD8^+^ T cells and CD19^+^ B cells were isolated using positive selection with CD3, CD4, CD8 or CD19 microbeads. CD4^+^CD45RA^+^ or CD4^+^CD45RO^+^ T-cell subsets were purified by using EasySep® Human Naïve CD4^+^ T Cell Enrichment Kit (StemCell Technologies, Vancouver, Canada) as previously described. Purity was checked by FACS analysis and was typically greater than 95%. Although we isolated T cells by positive selection, the selection procedure had a negligible effect on the T cell activation as CD25 and CD69 expression did not change.

### Flow cytometry

1×10^6^ cells were washed in phosphate-buffered saline (PBS) containing 0.5% bovine serum albumin (BSA) and incubated with specific fluorochrome-conjugated antibodies and analyzed on a FACSCalibur flow cytometer (Becton-Dickinson). For membrane-bound TGF-β, mononuclear cells freshly-isolated from lymphoma patients or lymphoma cell lines were stained with PE-conjugated anti-LAP/TGF-β1 (Biolegend, San Diego, CA) and analyzed by flow cytometry.

Transcriptional factor Foxp3 expression was determined by flow-based intracellular staining following the manufacturer's instructions. Cells were fixed and permeabilized with reagents from Foxp3 staining kit (Biolegend, San Diego, CA). Cells were then stained with fluorochrome-conjugated antibody against Foxp3 plus fluorochrome-conjugated anti-CD4 antibody for 30 min and analyzed by flow cytometry. Acquired data were analyzed by using FlowJo (Tree Star, Ashland, OR), WinMDI 2.9 (TheScripps Research Institute, SanDiego, CA) or Flowing Software 2 (Cell Imaging Core, Turku Centre for Biotechnology).

### Cytokine intracellular staining

Cells were washed and subjected to fixation, permeabilization, staining with fluorochrome-conjugated antibodies against IL-2, IL-17, IFN-γ and analysis by flow cytometry. For T_H_17 cell induction, we cultured CD4^+^ T cells in anti-CD3-coated plates with either IL-6 (10 ng/ml) plus IL-1β (10 ng/ml) or IL-23 (10 ng/ml) in the presence or absence of either IL-2 or anti-IL-2 or anti-IL-2Rα or β for 3 days. IL-17 or IFN-γ expression was measured by intracellular staining after cells were re-stimulated with phorbol myristate acetate and ionomycin (PMA/Ion) plus Brefeldin A for 4 h.

### Proliferation assays

T cell proliferation was measured by CFSE staining. Briefly, CD4^+^ or CD8^+^ T cells were stained with CFSE (5 μM) and cultured on anti-CD3 coated plates in the presence or absence of TGF-β. Cells were harvested at day 3 and analyzed on a flow cytometer.

### ELISA assay

The concentration of TGF-β or IL-2 in serum or culture supernatants was measured by ELISA (R&D Systems, Minneapolis, MN) according to the manufacturer's instructions. To activate latent TGF-β to the immunoreactive form, serum or supernatants were acidified and neutralized by incubating with 1N HCl and then 1.2N NaOH/0.5 M HEPES. The optical density of each well was determined using a SpectraMax190 microplate reader (Molecular Devices, Sunnyvale, CA) set to 450 nm and analyzed using SoftMax Pro 5 software. A standard curve was generated to calculate the concentration of TGF-β for each set of samples assayed. To control for the TGF-β concentration in the culture medium, we set a well of culture medium without cells and used it as a baseline to be subtracted for all samples assayed. For some experiments, a serum-free culture medium, in which we demonstrated TGF-β to be completely absent (Opti-MEM, Life Technologies, Grand Island, NY), was used to measure TGF-β concentration in cultured cells.

### TGF-β binding and co-culture

Lymphoma cell lines or freshly-isolated lymphoma B cells were suspended in PBS and incubated with or without 100 ng/ml TGF-β (PeproTech) on ice for 30 min. After extensive washing, cells were pretreated with 5 μg/mL mouse IgG on ice for 30 minutes (blocking) and then stained with biotinylated anti-TGF-β Ab (2 μg/mL) and PE-streptavidin. Membrane-bound TGF-β was measured by flow cytometry. Binding was assessed and expressed as mean fluorescence intensity (MFI). For heparin blocking experiments, 10 μg/ml heparin was incubated with TGF-β for 30 min and added into an OCI-Ly19 cell suspension. For TGF-β receptor blocking experiments, OCI-Ly19 cells were pretreated with 5 μg/mL anti-TGF-β type I or II receptor Ab on ice for 30 min before incubation with TGF-β. For stripping, TGF-β-pretreated OCI-Ly19 cells were incubated with pH 4.0 NaCl/citrate for 45 seconds. After washing, TGF-β untreated or pre-treated OCI-Ly10 or OCI-Ly19 cells with or without stripping were co-cultured with CFSE-labeled CD4^+^ T cells in an anti-CD3 Ab-coated plate in the presence of anti-CD28 Ab at a ratio of 1:1 or 5:1 for 3 days. Cells were harvested at day 3 and analyzed on a flow cytometer.

### Statistical analysis

Statistical analysis was performed using Student's t test. Significance was determined at p<0.05.

## Results

### Soluble TGF-β expression in B-cell NHL

**Figure 1:**
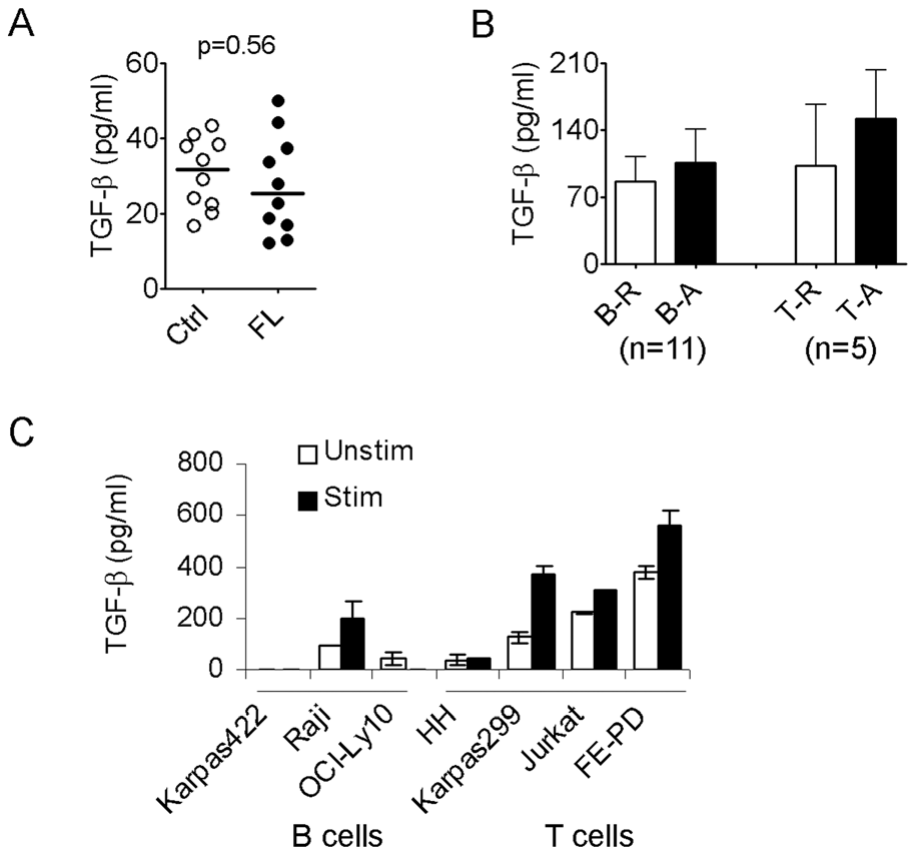
Soluble TGF-β expression in B-cell NHL. (A) TGF-β serum levels measured by ELISA in untreated FL patients (mean: 27.67+/−13.2, n = 10) and healthy donors (median: 30.76+/−9.47 pg/ml, n = 10; p = 0.56). (B) TGF-β levels in culture supernatants of freshly-isolated malignant B (B, n = 11) and intratumoral T cells (T, n = 5) from B-cell NHL treated with (A) or without (R) PMA/Ion measured by ELISA. (C) A representative graph showing TGF-β levels in culture supernatants of B and T cell lines treated with (Stim) or without (Unstim) PMA/Ion measured by ELISA (n = 3).

To explore the role of TGF-β in the regulation of tumor immunity in B-cell NHL, we measured serum levels of TGF-β from patients with untreated follicular lymphoma (FL) and healthy individuals. While we detected TGF-β in serum from both FL patients and healthy individuals, no significant difference in levels of TGF-β between the two groups was observed. The mean TGF-β1 level (+/− standard deviation) in untreated FL patients was 27.67 pg/ml with the range from 12.2 to 49.9 pg/ml (+/− 13.2, n = 10) compared to 30.76 pg/ml in healthy controls with the range from 16.7 to 43.4 pg/ml (+/− 9.47, n = 10; p = 0.56) (Figure 1A). We next measured the concentration of TGF-β by ELISA in supernatants of cultured malignant B and intratumoral T cells from lymphoma biopsy specimens. As shown in Figure 1B, both lymphoma B cells (median: 62.6, range from undetectable to 230, n = 11) and intratumoral T cells (median: 72.7; range from undetectable to 350, n = 5) were able to produce TGF-β at variable levels among patient samples. Activation with PMA/Ion slightly increased TGF-β production by both lymphoma B and intratumoral T cells. We also measured the concentration of TGF-β in culture medium of lymphoma B and T cell lines. As with patient cells, we found that TGF-β is variably produced by the different lymphoma B and T cell lines. Treatment of these cells with PMA/Ion enhanced TGF-β secretion (Figure 1C). These results indicate a potential role of soluble TGF-β in B cell NHL.

### Effect of TGF-β on the differentiation of intratumoral T cells in B-cell NHL

**Figure 2:**
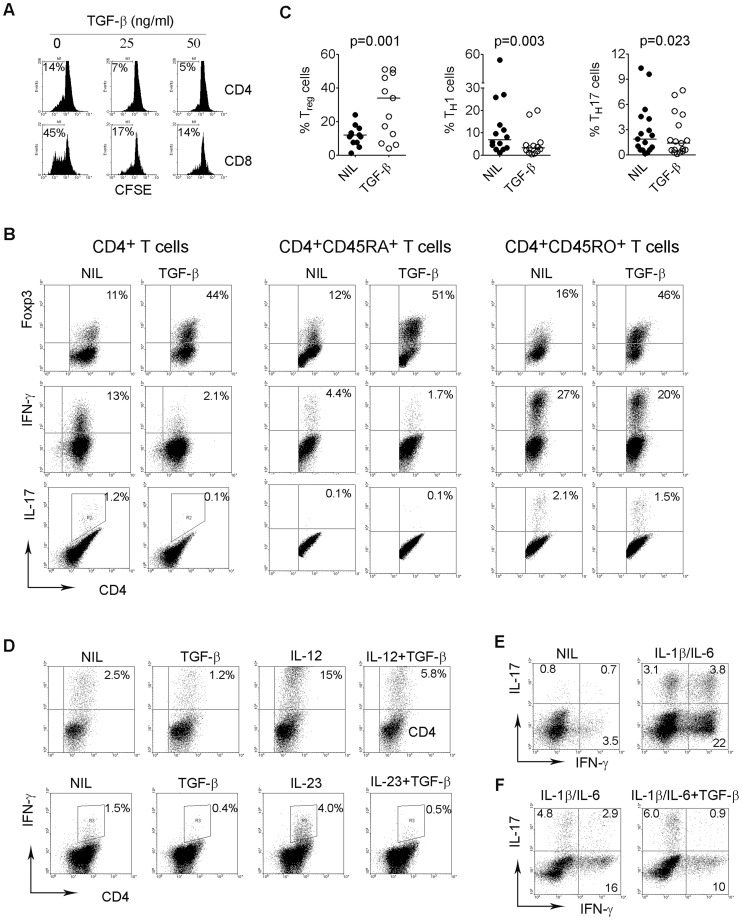
Effect of TGF-β on the differentiation of intratumoral T cells in B-cell NHL. (A) Representative histograms (n = 3) showing proliferation measured by CFSE staining of CD4^+^ or CD8^+^ T cells treated with different doses of TGF-β. Proliferative capacity was expressed by calculating the number of CFSE^dim^ cells. (B) Representative dot plots showing the expression of Foxp3, IFN-γ or IL-17 in CD4^+^, CD4^+^CD45RA^+^ or CD4^+^CD45RO^+^ T cells treated with or without TGF-β. (C) Summary of the numbers of T_reg_ (CD4^+^Foxp3^+^), T_H_1 (CD4^+^IFN-γ^+^) or T_H_17 (CD4^+^IL-17^+^) cells induced by TGF-β. (D) Representative dot plots (n = 3) showing the expression of IFN-γ in CD4^+^ T cells treated with or without TGF-β or IL-12 or IL-23 alone or in combination. (E) Representative dot plots (n = 10) showing the expression of IL-17 in CD4^+^ T cells treated with or without IL-1β plus IL-6. (F) Representative dot plots (n = 6) showing the expression of IL-17 in CD4^+^ T cells treated with or without TGF-β in the presence of IL-1β plus IL-6.

TGF-β is a pleiotropic cytokine involved in cell differentiation and apoptosis of a variety of cell types. However, little is known about the effects of soluble TGF-β on intratumoral T cells in B-cell NHL. Because we observed significant secretion of TGF-β by some malignant B cells, we were interested in assessing the effects of TGF-β on intratumoral T cells. Using biopsy specimens from patients with B-cell NHL, we observed that TGF-β profoundly inhibits the proliferation of both CD4^+^ and CD8^+^ T cells stimulated by anti-CD3 and CD28 Abs (Figure 2A).

To test whether TGF-β was involved in the differentiation of T cells, we determined the effect of TGF-β on the generation of T_H_1 (CD4^+^IFN-γ^+^), T_H_17 (CD4^+^IL-17^+^) and T_reg_ (CD4^+^Foxp3^+^) cells in lymphoma specimens. Using different subsets of T cells isolated from biopsy specimens, we found that TGF-β enhances Foxp3 expression in CD4^+^, CD4^+^CD45RA^+^ naïve or CD4^+^CD45RO^+^ memory intratumoral T cells. In contrast, TGF-β suppressed the expression of IFN-γ and IL-17 in CD4^+^, CD4^+^CD45RA^+^ naïve or CD4^+^CD45RO^+^ memory intratumoral T cells (Figure 2B). Data acquired from multiple NHL samples (n = 12) indicated that TGF-β significantly promoted the development of T_reg_ cells and down-regulated the generation of T_H_1 and T_H_17 cells in the tumor microenvironment (Figure 2C).

IL-12 is a cytokine known to promote T_H_1 cell development. Similarly, IL-23 has been shown to enhance IFN-γ production in CD4^+^ T cells. We next tested whether TGF-β is able to suppress IL-12- or IL-23-mediated T_H_1 cell development. As shown in Figure 2D, both IL-12 and IL-23 were able to increase IFN-γ expression. In the presence of TGF-β, however, such effects were markedly attenuated. Because IL-1β together with IL-6 has been demonstrated to promote T_H_17 cell development, we then determined the effect of TGF-β on IL-1β/IL-6-mediated T_H_17 cell development. As shown in Figure 2E, we observed that treatment with IL-1β/IL-6 not only increases the numbers of CD4^+^ IL-17-producing cells, but also induces IL-17 expression in IFN-γ-producing cells. However, treatment with TGF-β decreased IFN-γ expression in CD4^+^ IL-17-producing T-cells induced by IL-1β/IL-6 (Figure 2F).

### Effects of TGF-β on IL-2 production by T cells

**Figure 3:**
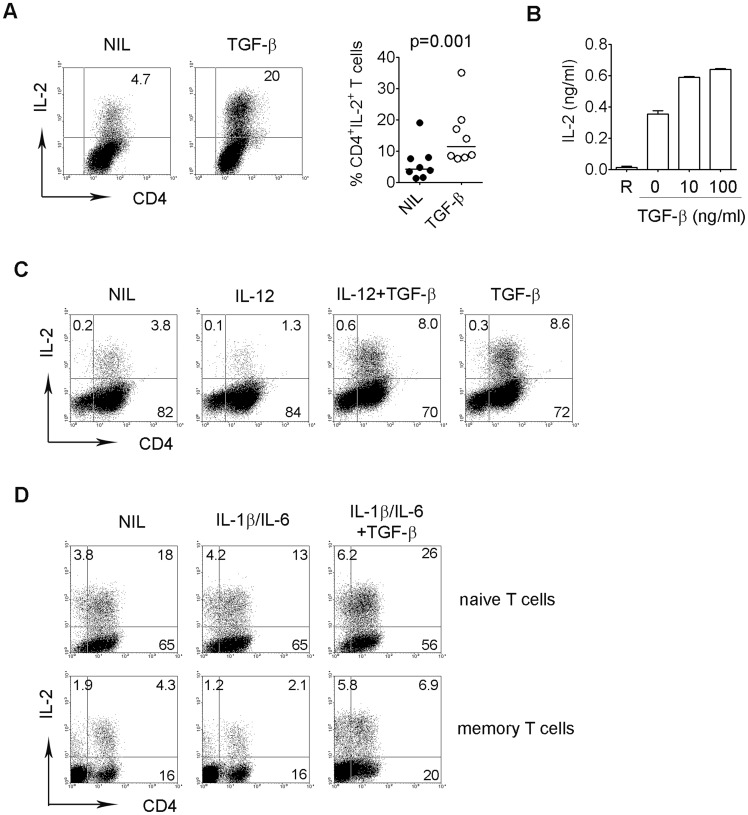
Effects of TGF-β on IL-2 production in T cells. (A) Representative dot plots showing the expression of IL-2 in CD4^+^ T cells treated with or without TGF-β. Data acquired from multiple samples were summarized (right, n = 8). (B) IL-2 levels measured by ELISA in culture supernatants of freshly-isolated intratumoral T cells cultured in anti-CD3-coated plate with escalating doses of TGF-β (n = 3). R: resting T cells. (C) Representative dot plots (n = 3) showing the expression of IL-2 in CD4^+^ T cells treated with or without IL-12 or TGF-β alone or in combination. (D) Representative dot plots (n = 5) showing the expression of IL-2 in CD4^+^ CD45RA^+^ naïve or CD4^+^CD45RO^+^ memory T cells treated with or without TGF-β in the presence of IL-1β plus IL-6.

We have previously shown that IL-2 signaling is crucial to T cell differentiation by inhibiting effector (T_H_1, T_H_17) cells and promoting T_reg_ cells. It has been shown that IL-2 signaling plays an essential role in TGF-β-mediated induction of T_reg_ cells. Therefore, we first determined whether TGF-β had an effect on IL-2 production by intratumoral CD4+ T cells and found that TGF-β up-regulates IL-2 expression in intratumoral CD4+ T cells (Figure 3A). Similarly, the IL-2 concentration in culture supernatants was elevated when CD4+ T cells were treated with TGF-β (Figure 3B).

To further confirm TGF-β-mediated induction of IL-2 production, we analyzed the effect of TGF-β on the regulation of IL-2 expression by cytokines that are able to influence the development of T_H_1 or T_H_17 cells, specifically IL-12, IL-1β and IL-6. We found that IL-2 production was down-regulated when intratumoral CD4^+^ T cells were treated with IL-12. However, in the presence of TGF-β, the effect of IL-12 was not only overcome, but IL-2 production by CD4^+^ cells actually increased nearly 5-fold (Figure 3C). IL-1β plus IL-6 modestly suppressed IL-2 production by intratumoral CD4^+^ T cells. However, treatment with TGF-β significantly increased IL-2 production in both naïve and memory cells even in the presence of IL-1β plus IL-6 (Figure 3D). These results suggest that TGF-β may play a major role in IL-2 production in the tumor microenvironment in NHL.

### Effects of TGF-β and IL-2 signaling in the regulation of T cell differentiation in B-cell NHL

**Figure 4:**
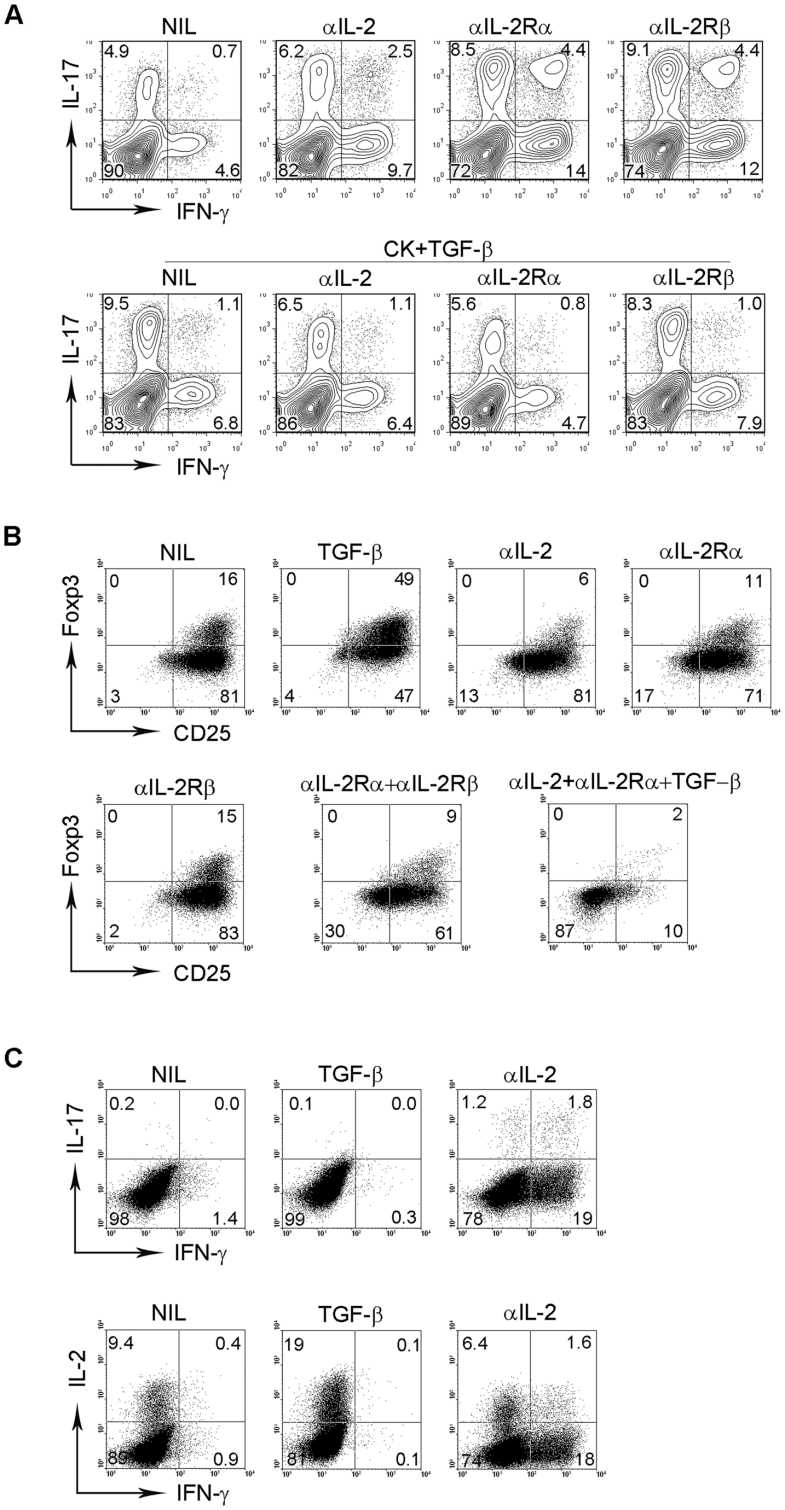
Effects of IL-2 signaling on TGF-β-mediated regulation of T cell differentiation in B-cell NHL. (A) Representative dot plots (n = 6) showing the expression of IFN-γ and IL-17 in CD4^+^ T cells treated with or without αIL-2 or αIL-2Rα or β in the presence or absence of TGF-β plus IL-6 and IL-23. (B) Representative dot plots (n = 3) showing the expression of Foxp3 and CD25 in CD4^+^ T cells treated with or without TGF-β, αIL-2 or αIL-2Rα or β alone or in combination. (C) Dot plots from a representative sample (n = 5) showing the expression of IFN-γ, IL-17 and IL-2 in CD4^+^ T cells treated with or without TGF-β or αIL-2.

Given that TGF-β up-regulates IL-2 production, we wanted to test whether IL-2 signaling plays a role in TGF-β-mediated regulation of T cell differentiation in the lymphoma microenvironment. As shown in Figure 4A, treatment with blocking antibodies against IL-2, IL-2Rα or IL-2Rβ up-regulated IFN-γ or IL-17 in CD4^+^ T cells, consistent with our previous reports that IL-2 signaling plays an important role in regulating T-cell differentiation [21], [29]. Notably, interruption of IL-2 signaling induced co-expression of IFN-γ and IL-17 in CD4^+^ T cells and formation of a CD4^+^IFN-γ^+^IL-17^+^ population. However, the addition of TGF-β to IL-6 and IL-23 down-regulated expression of IFN-γ and IL-17 in CD4^+^ T cells treated with antibodies against IL-2 or IL-2Rα or IL-2Rβ. The CD4^+^IFN-γ^+^IL-17^+^ population induced by interruption of IL-2 signaling was not detected in the presence of TGF-β, IL-6 and IL-23. This further suggests that TGF-β plays a role in polarizing IL-17-producing cells into a T_H_17 subset similar to that originally described in mice.

In addition to T_H_ cells, TGF-β is also involved in IL-2-mediated T_reg_ cell development. As shown in Figure 4B, TGF-β up-regulated Foxp3 expression in CD4^+^ T cells as expected. Treatment with blocking antibodies against either IL-2, IL-2Rα, or IL-2Rβ alone or in combination significantly down-regulated Foxp3 expression. Importantly, TGF-β failed to up-regulate Foxp3 expression in the absence of IL-2 signaling and further decreased Foxp3 and CD25 expression in CD4^+^ T cells co-treated with antibodies against IL-2 and IL-2Rα. These results indicate that IL-2 signaling plays a critical role in TGF-β-mediated T_reg_ cell development.

Next, we determined whether a reverse correlation could be observed between production of IL-2 and either IFN-γ or IL-17 expression in CD4^+^ T cells. As shown in Figure 4C, we found that an increase in IFN-γ and IL-17 expression in response to blockade of IL-2 signaling by anti-IL-2 Ab correlates with a decrease in IL-2 production. In contrast, an increase in IL-2 production in response to TGF-β was associated with suppressed expression of IFN-γ or IL-17 in CD4^+^ T cells. This inverse correlation was observed repeatedly (n = 5), and these findings strongly suggest that TGF-β and IL-2 signaling play an essential role in T-cell differentiation.

### Expression of membrane-bound TGF-β and binding of TGF-β to cell surface of lymphoma cells

**Figure 5:**
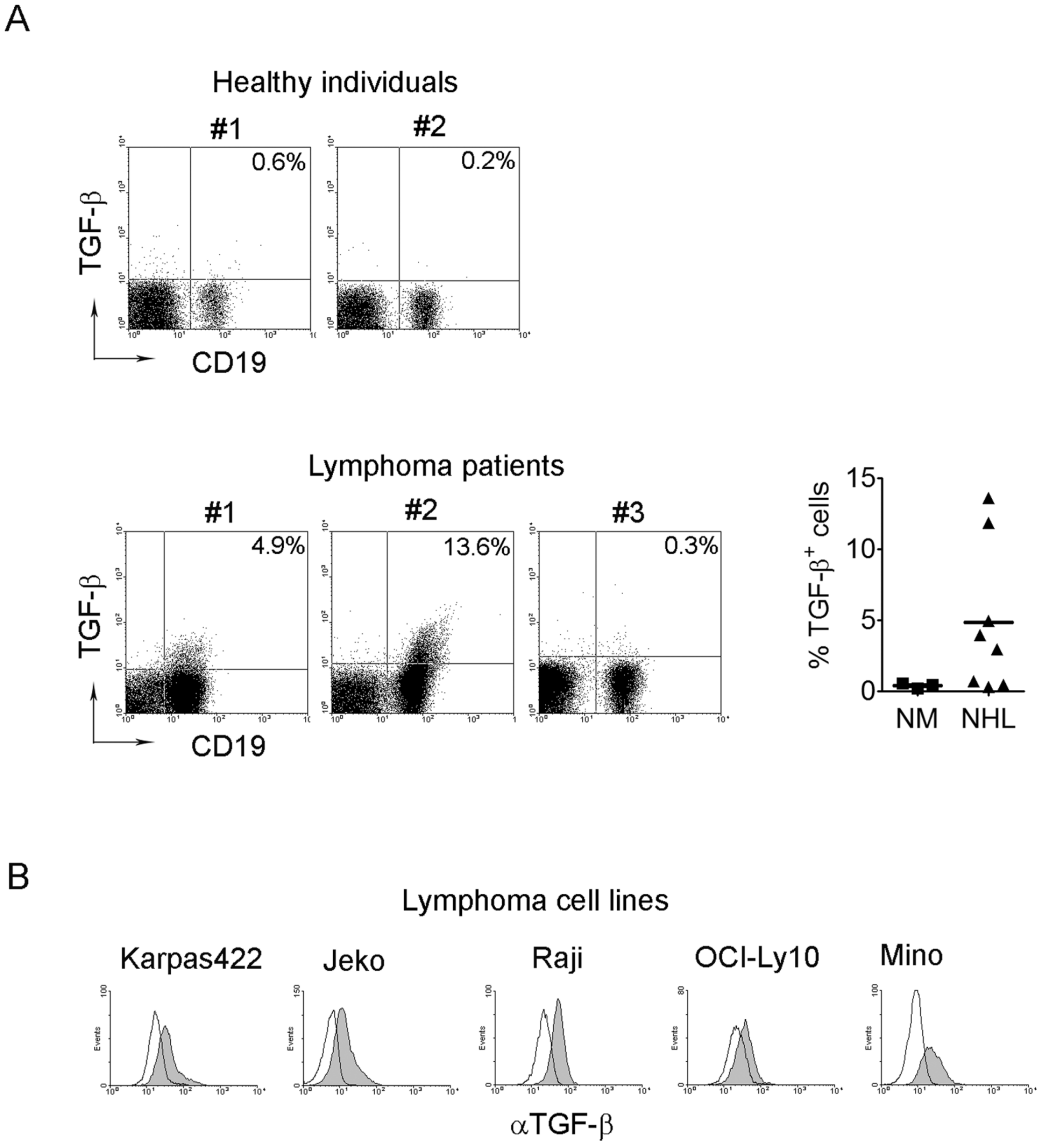
Membrane-bound TGF-β expression in B-cell NHL. (A) Representative dot plots showing the expression of membrane-bound TGF-β on CD19^+^ B cells from 2 healthy individuals (NM) and 3 lymphoma biopsy specimens (NHL). Data acquired from multiple samples were summarized (right). (B) Histograms showing the expression of membrane-bound TGF-β on malignant B cell lines measured by flow cytometry. Solid line indicates isotype control, the shaded indicates membrane-bound TGF-β expression.

In addition to the presence of soluble TGF-β, we were also interested in assessing the expression of membrane-bound TGF-β on the surface of cells in the tumor microenvironment of B-cell NHL. By using freshly-isolated mononuclear cells derived from biopsy specimens of patients with B-cell NHL, we found that membrane-bound TGF-β is only expressed on CD19^+^ lymphoma cells, although the number of TGF-β-expressing lymphoma cells varied among patient samples (Figure 5A). To confirm this finding, we screened a number of B cell lines and found that almost all lymphoma B cell lines tested express membrane-bound TGF-β on their cell surface (Figure 5B). Normal B cells from peripheral blood of healthy donors, however, had no expression of membrane-bound TGF-β (Figure 5A).

**Figure 6:**
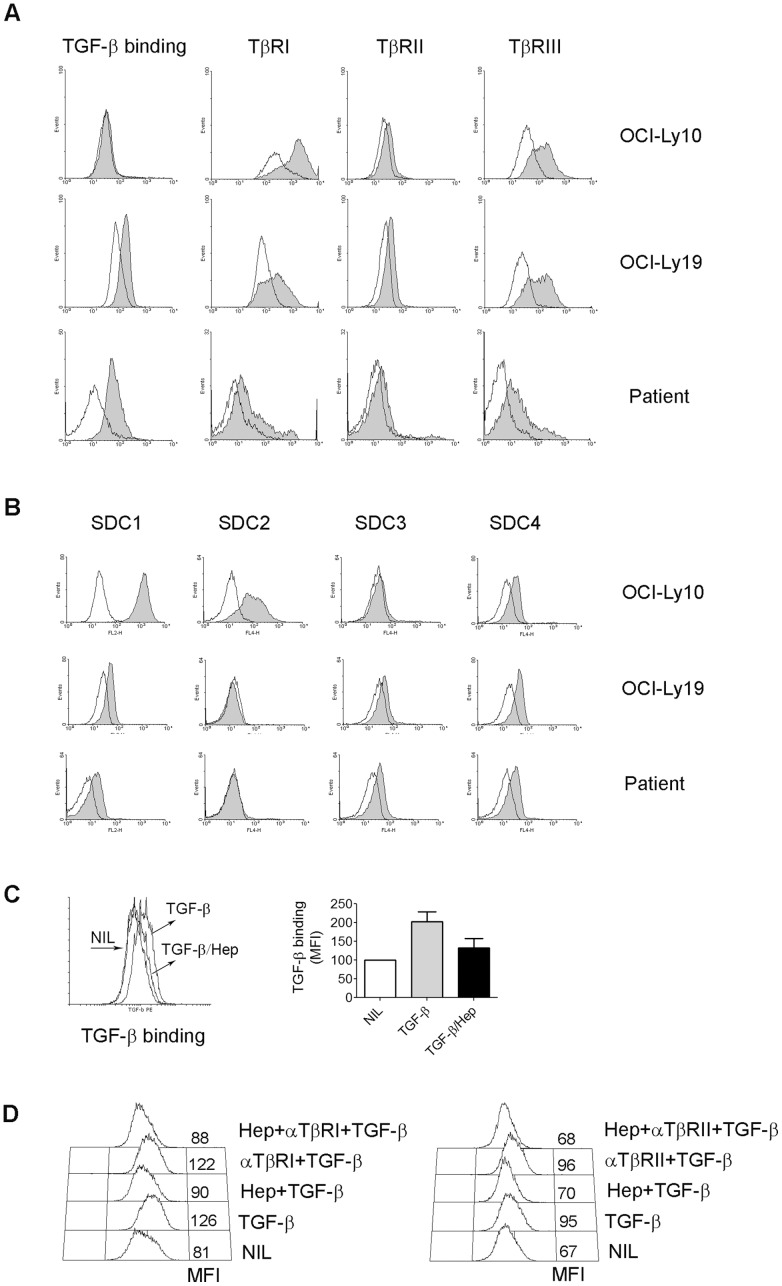
Lymphoma cells trap TGF-β through heparan sulfate. (A) Histograms showing TGF-β binding or TGF-β receptors (I, II or III) measured by flow cytometry on lymphoma B cell lines OCI-Ly10 and OCI-Ly19 or freshly-isolated CD19^+^ B cells from a representative lymphoma patient. (B) Histograms showing syndecans (SDC) measured by flow cytometry on lymphoma B cell lines or freshly-isolated CD19^+^ B cells from a representative lymphoma patient. (C) Representative histograms showing TGF-β binding to OCI-Ly19 cells pre-incubated with or without heparin (Hep). The binding capacity was expressed by mean fluorescent index (MFI) and summarized from multiple experiments (n = 7) in the graph on the right. (D) Representative histograms showing TGF-β binding to OCI-Ly19 cells pre-incubated with Hep or αTβRI (left) or αTβRII (right) alone or in combination. The binding capacity was expressed by MFI.

Given our finding that TGF-β was expressed on the cell surface of lymphoma B cells, we wanted to test whether membrane-bound TGF-β was involved in intratumoral T cell differentiation and function and if malignant B cells were able to trap TGF-β on the cell surface. To test this, we incubated lymphoma cell lines or freshly-isolated lymphoma B cells with TGF-β for 30 min and measured TGF-β binding by flow cytometry. As shown in Figure 6A, binding of TGF-β to lymphoma B cells was highly variable. We found that TGF-β binds to most lymphoma cell lines except Karpas 422 and OCI-Ly10 cells (Figure 6A, Table S2). Similarly, TGF-β was able to variably bind to freshly-isolated lymphoma B cells from biopsy specimens. In some specimens, TGF-β bound to lymphoma cells and in other specimens this binding was negligible (Figure 6A, Table S2). In contrast, binding of TGF-β was undetectable on B cells from peripheral blood of healthy donors (Table S2).

Next, we tested the underlying mechanism mediating TGF-β binding to lymphoma B cells. To do this, we first determined the expression of TGF-β receptors (TβRI, II and III) on primary lymphoma cells and cell lines. As shown in Figure 6A and Table S2, TβRIII was strongly expressed on most lymphoma cell lines. The expression levels of TβRI varied among cell lines. TβRII, the subunit primarily involved in TGF-β initial binding, was expressed at low or negligible levels on most lymphoma cell lines. In patient samples, the expression levels of all three receptor subunits of TGF-β were low or absent, consistent with findings that loss of TβRI or II expression is detectable in most types of common cancer [30]. These results indicate that other mechanisms exist and are involved in mediating the binding of TGF-β to lymphoma cells.

While receptor-mediated binding is usually the major mechanism of TGF-β binding to a cell's surface, TGF-β can bind to cells through heparan sulfate (HS) as well [31], [32]. In this regard, we tested whether HS was involved in TGF-β binding to malignant B cells. Syndecans (SDC), including family members 1, 2, 3 and 4, express abundant HS chains and are a major source for HS expression on the cell surface [33]. Therefore, we first determined the expression of SDC1-4 on lymphoma cells. As shown in Figure 6B and Table S3, we found that lymphoma cell lines and lymphoma B cells obtained from patients variably express different types of SDC.

Next, we measured whether heparin (Hep) blocked TGF-β binding to malignant B cells by using OCI-Ly19 cells that are capable of binding TGF-β. TGF-β was treated with heparin prior to incubation with OCI-Ly19 cells. We found that this pretreatment nearly abolishes TGF-β binding to OCI-Ly19 cells (Figure 6C–D). To further confirm that HS is involved in TGF-β binding to malignant B cells and not the TGF-β receptors, we determined whether blocking the TGF-β receptor would prevent TGF-β from binding to OCI-Ly19 cells in the same manner as HS. We found that pretreatment of TGF-β with anti-TβRI or II Ab is not able to prevent TGF-β from binding to OCI-Ly19 cells (Figure 6D), yet Hep prevents TGF-β from binding to OCI-Ly19 cells pre-treated either with or without anti-TβRI or II Ab. These results strongly suggest the involvement of HS in TGF-β binding to lymphoma cells.

### Effects of membrane-bound TGF-β on intratumoral T cell function

**Figure 7:**
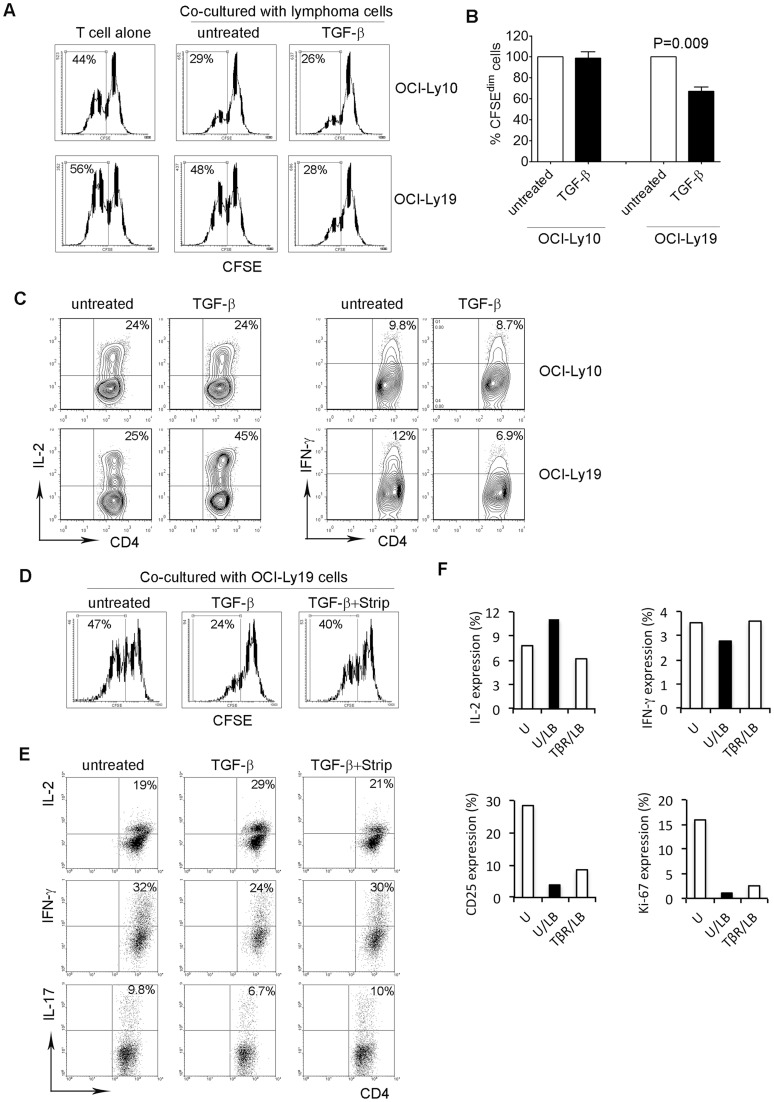
Effects of TGF-β-bearing lymphoma B cells on intratumoral T cell function. (A) Representative histograms showing proliferation of CD4^+^ T cells, measured by CFSE staining, co-cultured with untreated or TGF-β pre-incubated OCI-Ly10 or OCI-Ly19 cells. Proliferative capacity was expressed by calculating the number of CFSE^dim^ cells. (B) Summarized data showing proliferation of CD4^+^ T cells co-cultured with untreated or TGF-β pre-incubated OCI-Ly10 (n = 5) or OCI-Ly19 (n = 8) cells. Changes in proliferation of CD4^+^ T cells were expressed by percentage of CFSE^dim^ cells in TGF-β pre-incubated group over untreated group. (C) Representative dot plots (n = 3) showing the expression of IL-2 or IFN-γ measured by intracellular staining in CD4^+^ T cells co-cultured with untreated or TGF-β pre-incubated OCI-Ly10 or OCI-Ly19 cells. (D) Representative histograms (n = 3) showing proliferation of CD4^+^ T cells co-cultured with untreated, TGF-β pre-incubated OCI-Ly19 cells with or without stripping by low pH NaCl/citrate buffer measured by CFSE staining. Proliferative capacity was expressed by calculating the number of CFSE^dim^ cells. (E) Representative dot plots (n = 3) showing the expression of IL-2 or IFN-γ or IL-17 in CD4^+^ T cells co-cultured with untreated or TGF-β pre-incubated OCI-Ly19 cells with or without stripping by low pH NaCl/citrate buffer measured by intracellular staining. (F) Graphs from a representative lymphoma patient sample showing the expression of IL-2, IFN-γ, CD25 and Ki-67 in CD4^+^ T cells pre-treated with anti-TGF-βR (TβR) and then cocultured with lymphoma B cells (LB) in anti-CD3-coated plate for 3 days. The expression of IL-2, IFN-γ, CD25 and Ki-67 in CD4^+^ T cells was determined by flow cytometry and expressed as a percentage of the total cells. Untreated T cells (U) were used as a control.

We observed that lymphoma B cells specifically express membrane-bound TGF-β and found in vitro that TGF-β is able to bind to the surface of lymphoma B cells. We then wanted to test whether membrane-bound TGF-β on lymphoma B cells has a similar effect on T cell proliferation and cytokine production as soluble TGF-β. OCI-Ly10 or OCI-Ly19 cells were incubated with TGF-β for 30 min and then washed 3 times to remove residual TGF-β prior to co-culture with CD4^+^ T cells. As shown in Figure 7A–B, TGF-β-pretreated OCI-Ly19 cells, which possess strong TGF-β binding capacity, clearly inhibited the proliferation of intratumoral T cells compared to untreated OCI-Ly19 cells. The number of CFSE^dim^ T cells was significantly reduced when cocultured with OCI-Ly19 cells preincubated with TGF-β compared to untreated OCI-Ly19 cells. In contrast, we did not see a difference in proliferation of T cells (the number of CFSE^dim^ cells) cocultured with OCI-Ly10 cells preincubated with or without TGF-β, given that OCI-Ly10 cells are not able to bind TGF-β on the cell surface. In parallel with T cell proliferation, cytokine production in T cells was also influenced by co-culture with OCI-Ly19 cells pretreated with TGF-β (Figure 7C). We found that CD4^+^ T cells co-cultured with OCI-Ly19 cells pre-incubated with TGF-β have increased IL-2 and decreased IFN-γ production compared to CD4^+^ T cells co-cultured with untreated OCI-Ly19 cells. Again, there was no difference in IL-2 and IFN-γ production in CD4^+^ T cells co-cultured with OCI-Ly10 cells pre-incubated with TGF-β compared to untreated OCI-Ly10 cells (Figure 7C). These results are consistent with our observation that TGF-β suppresses T cell proliferation and regulates cytokine production in CD4^+^ T cells. Furthermore, these findings clearly show that TGF-β bound to the lymphoma cell surface is functionally active.

To further confirm the involvement of membrane-bound TGF-β in lymphoma cell-mediated regulation of T cell function, we determined whether stripping TGF-β off the lymphoma cell surface reversed the inhibition of T cells. As shown in Figure 7D, OCI-Ly19 cells pre-incubated with TGF-β inhibited the proliferative capacity of CD4^+^ T cells compared to untreated OCI-Ly19 cells. However, when the OCI-Ly19 cells were treated with a stripping solution, the inhibition of T cell proliferation by TGF-β treated OCI-Ly19 cells was attenuated. Similarly, production of IL-2, IFN-γ or IL-17 production in CD4^+^ T cells by TGF-β pre-incubated OCI-Ly19 cells was restored when the CD4^+^ T cells were co-cultured with stripped TGF-β pre-treated OCI-Ly19 cells (Figure 7E). These results strongly suggest that membrane-bound TGF-β is involved in OCI-Ly19 cell-mediated inhibition of T cell function.

Given that lymphoma B cells constitutively express TGF-β on cell surface, we wanted to know whether this expression has biological relevance as regards the function of intratumoral T cells. To test this, we determined the effect of TGF-β expressing lymphoma cells on freshly-isolated intratumoral T cells preincubated with anti-TGF-β receptor (I and II) Abs. Activation (CD25 and Ki-67 expression) and cytokine production (IL-2 and IFN-γ) of intratumoral T cells were measured by flow cytometry. As shown in Figure 7F, when cocultured with lymphoma B cells, T cells pretreated with anti-TGF-β receptor (I and II) Abs displayed increased expression levels of CD25 and Ki-67 compared to untreated cells. In regards to cytokine production, we observed that blockade of TGF-β signaling using an anti-TGF-β receptor Ab results in the restoration of IFN-γ-expressing cells as well as a decrease in the number of IL-2^+^ T cells. The finding that TGF-β signaling blockade attenuates lymphoma cell-mediated effects on intratumoral T cells indicates that TGF-β expression on the cell surface of lymphoma B cells plays an important role in affecting the function of intratumoral T cells in B-cell NHL.

## Discussion

Although TGF-β has been intensively investigated in a variety of tumor types including B-cell NHL, studies have focused on the effects of TGF-β on the malignant cells and very few studies have explored the effects of TGF-β on T cell differentiation and function in B-cell NHL. We have previously shown that a very immunosuppressive tumor microenvironment is present in B-cell NHL, but the underlying mechanisms for this suppressive microenvironment are only partially understood. In this study, we have presented evidence that both soluble and membrane-bound TGF-β are significantly involved in the regulation of intratumoral T cells by promoting T_reg_ cell generation and inhibiting effector T_H_ cells thereby contributing to the suppressive tumor microenvironment in B-cell NHL.

TGF-β is synthesized as a prepro-TGF-β precursor and is secreted after being processed in the Golgi apparatus as a soluble form. Subsequent studies have shown TGF-β to also be expressed on the cell surface as a membrane-bound form [5]. Different types of cells under a variety of pathophysiological conditions can express TGF-β, and our data show that TGF-β is produced by both malignant B and intratumoral T cells. Interestingly, while a majority of studies found that membrane-bound TGF-β is expressed by T cells, most notably T_reg_ cells, we observed that it was lymphoma B cells and not other cells that express membrane-bound TGF-β in biopsy specimens from lymphoma patients. We therefore explored whether T cell function and differentiation were regulated by lymphoma B cells with or without expression of membrane-bound TGF-β.

Recent studies have established that TGF-β plays a central role in mediating T cell differentiation by enhancing T_reg_ cell development and inhibiting effector T_H_ cell differentiation. As expected, we found that TGF-β promotes T_reg_ cell generation and inhibits effector T_H_ cell development in the lymphoma microenvironment. However, our data using human samples differ from the findings in mice with regards to the effects of TGF-β on T_H_17 cell development. While it has been demonstrated that TGF-β plus IL-6 promotes T_H_17 cells in mice, we observed that TGF-β had a suppressive, and not stimulating, effect on T_H_17 cell differentiation in B-cell NHL, a finding consistent with other previous reports [34]. In the presence of cytokines that have been demonstrated to promote T_H_17 cells in humans (IL-1β, IL-6 or IL-23 alone or in combination), TGF-β orchestrates T_H_17 development. It appeared that TGF-β blocked the expression of IFN-γ in IL-17-producing cells induced by IL-1β/IL-6 and “purified” IL-17-producing cells, polarizing them into a T_H_17 subset similar to that originally described in mice. While T cells treated with TGF-β alone inhibited IL-17 production (Figure 2B–C), the presence of IL-1β/IL-6 attenuated the inhibition, and the total number of IL-17-producing T cells remained similar between these two groups (Figure 2F). Taken together, these results confirm that TGF-β may play an important role in intratumoral T cell differentiation.

We also explored the underlying mechanism by which TGF-β mediates the regulation of T cell differentiation in B-cell NHL and found that IL-2 signaling was critically involved. Firstly, we found that TGF-β induces production of IL-2 in intratumoral T cells, which plays an essential role in mediating T cell differentiation. Secondly, we observed that the blockade of IL-2 signaling, by either blocking IL-2 or IL-2R, reverses TGF-β mediated regulation of T cell differentiation. These findings are consistent with previous reports that IL-2 signaling promotes the development of T_reg_ cells and inhibits the generation of T_H_17 cells [35]–[37].

However, regarding the effects of TGF-β on IL-2 production, the data remain controversial. A few studies have found that TGF-β inhibits T-cell proliferation, but this effect is associated with either decreased IL-2 production [38] or no effect on IL-2 secretion [39]. In contrast, we and others have found that TGF-β actually enhances IL-2 secretion, and in some studies, the increase in IL-2 is associated with an increase in transcriptional factors such as NFAT [40], [41]. While doses of TGF-β and culture conditions could account for this discrepancy, the finding that TGF-β inhibits IL-2 production [38], [39] does not appear to fit well with the universally demonstrated findings that TGF-β promotes the development of T_reg_ cells and inhibits generation of T_H_17 cells. Instead, these effects are more compatible with increased IL-2 levels and subsequent IL-2 signaling.

Because we found that membrane-bound TGF-β is specifically expressed on the surface of malignant B cells in B-cell NHL, we tested whether malignant B cells were able to trap TGF-β on their cell surface in an *in vitro* model. Indeed, TGF-β was capable of binding to the surface of primary lymphoma B cells and cell lines, although the binding capacity varied. While TGF-β receptors can mediate this binding, we and others [42]–[44] found that TGF-β receptors, especially receptor type II, are expressed at low or negligible levels on malignant B cells, suggesting that other mechanisms may account for TGF-β binding. This hypothesis is supported by the finding that treatment with a TGF-β receptor blocking Ab fails to prevent TGF-β binding to lymphoma cells [18]. In fact, TGF-β binding is affected by a number of molecules other than TGF-β receptor such as decorin, biglycan, and yet-to-be-identified TGF-β-binding cell surface proteins [45]–[47]. Previous studies have shown that TGF-β can bind to the surface of cells through HS [31], [32]. We found that HS is involved in TGF-β binding as pre-incubation with heparin blocked TGF-β binding to malignant B cells. Furthermore, we found that at least one type of syndecan, a major source of HS, is expressed on malignant B cells. The biological role of HS-mediated TGF-β binding is to protect TGF-β from proteolytic degradation [48]. This is particularly important in the tumor microenvironment because HS retains active TGF-β on the malignant cell surface. Indeed, we observed that TGF-β bound to or expressed on the surface of malignant B cells remains active and functions in a similar fashion to its soluble form by suppressing T cell proliferation and regulating T cell differentiation.

The composition of the tumor microenvironment and the mechanism of immunosuppression may differ in different histologies of B-cell NHL. To account for this, we used specimens from adequate numbers of patients with the common lymphoma subtypes and did not see a significant difference in terms of TGF-β expression and TGF-β-mediated suppression. Taken together, our results reveal a novel role for both soluble and membrane-bound TGF-β in the development and regulation of intratumoral T cells in B-cell NHL. We also report here on the ability of malignant B cells to trap TGF-β on their cell surface through an interaction with HS, thus modulating the immune response in the tumor microenvironment. This results in the suppression of intratumoral T cell proliferation and promotion of a regulatory T cell phenotype.

## Supporting Information

Table S1 (DOCX)

Table S2 (DOC)

Table S3 (DOC)
