# Supplementary material for: Soluble and Membrane-Bound TGF-β-Mediated Regulation of Intratumoral T Cell Differentiation and Function in B-Cell Non-Hodgkin Lymphoma
Source: PLoS One. 2013 Mar 15;8(3):e59456. doi: 10.1371/journal.pone.0059456 (PMC3598706; doi:10.1371/journal.pone.0059456)
Supplement: Table S1 — (DOCX) [file pone.0059456.s001.docx]

Table S1: Specimens used in the analysis of TGFβ

Biopsy specimens from lymphoma patients n = 35

Follicular lymphoma 10

Marginal zone lymphoma 9

Hodgkin lymphoma 5

Mantle cell lymphoma 4

Diffuse large cell lymphoma 3

Small lymphocytic lymphoma 3

T-cell lymphoma 1

Peripheral blood from lymphoma patients n = 9

Follicular lymphoma 6

Mantle cell lymphoma 2

Diffuse large cell lymphoma 1

Normal controls n = 14

Spleen 9

Lymph nodes 3

Peripheral blood 2
