# Supplementary material for: Soluble and Membrane-Bound TGF-β-Mediated Regulation of Intratumoral T Cell Differentiation and Function in B-Cell Non-Hodgkin Lymphoma
Source: PLoS One. 2013 Mar 15;8(3):e59456. doi: 10.1371/journal.pone.0059456 (PMC3598706; doi:10.1371/journal.pone.0059456)
Supplement: Table S2 — (DOC) [file pone.0059456.s002.doc]

Table S2: TGF-β binding and receptor expression on CD19^+^ B cells

| Cells | TGF-β binding | TGF-β receptors | | |
| --- | --- | --- | --- | --- |
|  |  | I | II | III |
| DoHH2 | + | - | + | +++ |
| Karpas 422 | - | ++ | +/- | +++ |
| OCI-Ly10 | - | ++ | + | +++ |
| OCI-Ly19 | ++ | + | + | +++ |
| Raji | + |  | - | + |
| SuDHL1 |  | ++ |  |  |
| BCMW |  | ++ |  |  |
| MMCL |  | ++ |  |  |
| OCI-Ly3 |  | - |  |  |
| Mino | + |  |  |  |
| Jeko | + |  |  |  |
| P1 | ++ |  |  |  |
| P2 | + |  |  |  |
| P3 | ++ |  |  |  |
| P4 | + | + | +/- | ++ |
| P5 | +/- | - | +/- | +/- |
| P6 | ++ | + | +/- | +/- |
| P7 | + | + | + | ++ |
| P8 | +/- | - | +/- | + |
| NM1 | - |  |  |  |
| NM2 | - |  |  |  |
| NM3 | - |  |  |  |

Note: P: patient; NM: healthy individual; -: non-detectable; +: detectable with low intensity compared to isotype control; ++: detectable with moderate intensity compared to isotype; +++: detectable with high intensity compared to isotype; blank: no test.
