# Supplementary material for: Soluble and Membrane-Bound TGF-β-Mediated Regulation of Intratumoral T Cell Differentiation and Function in B-Cell Non-Hodgkin Lymphoma
Source: PLoS One. 2013 Mar 15;8(3):e59456. doi: 10.1371/journal.pone.0059456 (PMC3598706; doi:10.1371/journal.pone.0059456)
Supplement: Table S3 — (DOC) [file pone.0059456.s003.doc]

Table S3: Syndecan expression on CD19^+^ B cells

| Cells | Syndecan | | | |
| --- | --- | --- | --- | --- |
|  | 1 | 2 | 3 | 4 |
| DoHH2 | + | - | -/+ | + |
| Karpas422 | +++ | +++ | + | + |
| OCI-Ly10 | +++ | +++ | - | ++ |
| OCI-Ly19 | + | - | + | + |
| SuDHL6 | ++ | - | + | ++ |
| Raji | + | -/+ | - | + |
| Jeko | - | - | +++ | + |
| Mino | +++ | +++ | ++ | ++ |
| RL | + |  |  | + |
| SuDHL4 | ++ |  |  | ++ |
| Pt1 | + |  |  | + |
| Pt2 | +++ |  |  | + |
| Pt3 | +/- | +/- | - | + |
| Pt4 | ++ | - | + | ++ |
| Pt5 | +/- | - | + | + |
| Pt6 | + | - | +/- | + |
| Pt7 | + | - | + | + |
| Pt8 | + | - | - | + |
| Pt9 | +/- | +/- | +/- | ++ |

Note: -: non-detectable; +: detectable with low intensity compared to isotype control; ++: detectable with moderate intensity compared to isotype; +++: detectable with high intensity compared to isotype; blank: no test.
